# Fingerprinting Proterozoic Bedrock in Interior Wilkes Land, East Antarctica

**DOI:** 10.1038/s41598-019-46612-y

**Published:** 2019-07-15

**Authors:** Alessandro Maritati, Jacqueline A. Halpin, Joanne M. Whittaker, Nathan R. Daczko

**Affiliations:** 1 Institute for Marine and Antarctic Studies, University of Tasmania, Private Bag 129, Hobart, TAS 7001 Australia; 2 ARC Centre of Excellence for Core to Crust Fluid Systems and GEMOC, Department of Earth and Planetary Sciences, Macquarie University, Sydney, NSW 2109 Australia

**Keywords:** Precambrian geology, Geophysics, Tectonics

## Abstract

Wilkes Land in East Antarctica remains one of the last geological exploration frontiers on Earth. Hidden beneath kilometres of ice, its bedrock preserves a poorly-understood tectonic history that mirrors that of southern Australia and holds critical insights into past supercontinent cycles. Here, we use new and recently published Australian and Antarctic geological and geophysical data to present a novel interpretation of the age and character of crystalline basement and sedimentary cover of interior Wilkes Land. We combine new zircon U–Pb and Hf isotopic data from remote Antarctic outcrops with aeromagnetic data observations from the conjugate Australian-Antarctic margins to identify two new Antarctic Mesoproterozoic basement provinces corresponding to the continuation of the Coompana and Madura provinces of southern Australia into Wilkes Land. Using both detrital zircon U–Pb–Hf and authigenic monazite U–Th–Pb isotopic data from glacial erratic sandstone samples, we identify the presence of Neoproterozoic sedimentary rocks covering Mesoproterozoic basement. Together, these new geological insights into the ice-covered bedrock of Wilkes Land substantially improve correlations of Antarctic and Australian geological elements and provide key constraints on the tectonic architecture of this sector of the East Antarctic Shield and its role in supercontinent reconstructions.

## Introduction

## Exploring Subglacial Geology in Interior Wilkes Land

The interior of Wilkes Land remains one of the most remote and unexplored sectors of the Precambrian East Antarctic Shield. Revealing ice-covered geology in this region is critical to understanding the assembly and breakup of the Nuna/Columbia, Rodinia and Gondwana supercontinents of which Wilkes Land was a centrepiece^1,2^. The bedrock also exerts important controls on the evolution of the Antarctic ice sheet^3^, underlying some of East Antarctica’s largest and most vulnerable glacial drainage basins (i.e. Vanderford, Totten, Moscow University Ice Shelf–MUIS) which together have the potential to contribute up to 3 m of global sea level rise in a warming climate^4^. However, due to thick ice cover and very limited rock exposure/sampling, our understanding of the age and composition of the Wilkes Land interior is inferred from the largely unconstrained projection of Australian geological counterparts into Antarctica^1^ and ages of detrital grains from glacio-marine sediments offshore of East Antarctica^5,6^.

**Figure 1:**
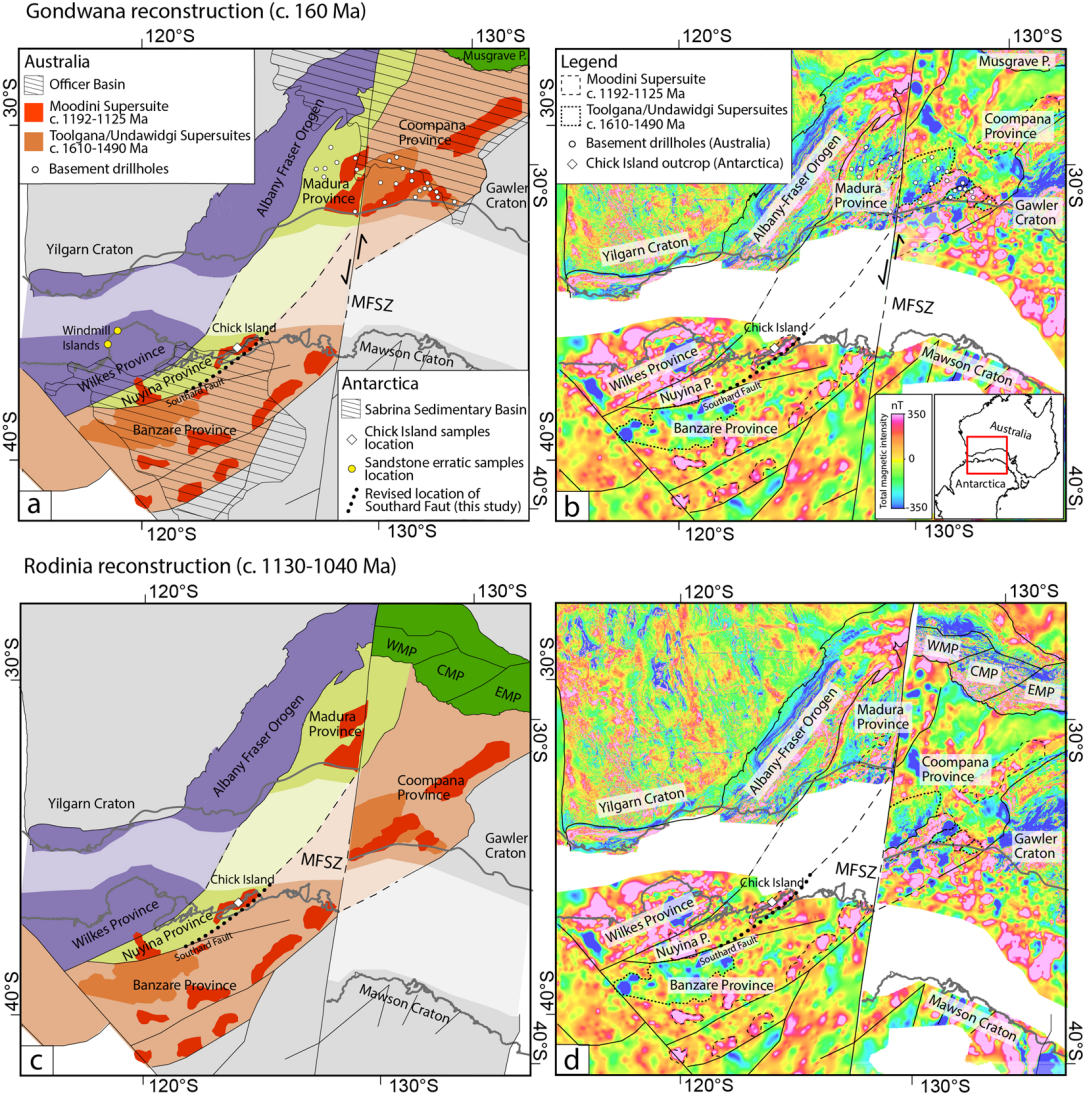
(Top) Reconstruction of (**a**) conjugate Proterozoic tectonic provinces and (**b**) total magnetic intensity (TMI) anomaly data of southwestern Australia and Wilkes Land in the Gondwana full-fit model of Aitken *et al*.^11^ with Australia fixed in its present-day reference frame. (Bottom) Reconstruction of (**c**) conjugate Proterozoic tectonic provinces and (**d**) total magnetic intensity (TMI) anomaly data of southwestern Australia and Wilkes Land in the late Mesoproterozoic reconstruction of the Rodinia configuration (c. 1130–1040 Ma) of Aitken *et al*.^7^ following retro-deformation of approximately 330 km of sinistral offset on the Mundrabilla-Frost Shear Zone. Australian tectonic elements are simplified from Raymond *et al*.^61^; interpretation of tectonic elements, geophysical lineaments and intrusive suites in Wilkes Land is from Aitken *et al*.^11^; simplified outline of the Sabrina Sedimentary Basin is adapted from Aitken *et al*.^3^; half arrows next to the Mundrabilla-Frost Shear Zone indicate sinistral displacement. Abbreviations are: CMP–central Musgrave Province; EMP–eastern Musgrave Province; MFSZ–Mundrabilla-Frost Shear Zone; WMP–West Musgrave Province.

Recent tectonic reconstructions of Gondwana that reconcile geological and geophysical signatures from the conjugate Australian and Antarctic plates revealed the presence of three large-scale Proterozoic basement provinces in Wilkes Land that accreted during supercontinent assembly^7^. The Wilkes province (Fig. 1a) occupies the region west of the Totten Glacier, including the well-studied outcrops of the Windmill Islands to the west of Law Dome^8,9^. This province has relatively robust links with two main peaks of magmatism and metamorphism (c. 1330–1260 Ma and c. 1220–1140 Ma) also documented in the Nornalup Zone of the Albany Fraser Orogen of southwestern Australia^10^. The Mawson Craton (East Mawson Craton of Aitken *et al*.^11^) comprises the Archean to Paleoproterozoic rocks in Terre Adélie and George V Lands and has geological affinities with the Gawler Craton of South Australia^2^ (Fig. 1a).

The intervening region (West Mawson Craton of Aitken *et al*.^11^), underlying most of the combined Vanderford-Totten-MUIS continental ice sheet, is only characterised by sparse legacy geological data^12,13^ and remains vastly underexplored. This region is interpreted to be the Antarctic extension of the unexposed Coompana and/or Madura provinces of southern Australia^7^ (Fig. 1a). Recent isotopic and geochemical data from drillhole samples in these Australian provinces fingerprint a distinctively juvenile isotopic character, similar to the central Australian Musgrave Province, that has been interpreted to represent c. 1950 Ma oceanic crust reworked by a series of accretionary tectonic events throughout the Mesoproterozoic, involving significant mantle input^14–16^. The Coompana Province is characterised by two dominant magmatic supersuites – the c. 1610 Ma Toolgana Supersuite with chemical and isotopic characteristics of primitive arc, and the rift-related c. 1490 Ma Undawidgi Supersuite^14,17,18^, while the Madura Province comprises a series of oceanic crustal assemblages that define oceanic subduction-related events between 1475 and 1389 Ma^19,20^. Both the Coompana and Madura provinces are extensively intruded by granitic rocks of the c. 1192–1125 Ma Moodini Supersuite^17–19^. The Coompana and Madura provinces are separated by the transcontinental Mundrabilla-Frost Shear Zone which experienced approximately 330 km sinistral offset during the last phases of Rodinia assembly (1130–1040 Ma)^7,20^ and extends into East Antarctica (Fig. 1a,c). While strike-slip movement resulted in the truncation of structural trends and the displacement of the original boundary between the two provinces in Australia, this shear zone cuts east of the conjugate crust of East Antarctica into the Mawson Craton (Fig. 1).

2D gravity modelling and depth to magnetic basement estimates also reveal that large areas of the interior Wilkes Land are blanketed by the ≥1 km-thick Sabrina Sedimentary Basin, covering an area of approximately 500,000 km^2,^^3^ (Fig. 1a). In Australia, parts of the Coompana and Madura provinces are covered by extensive sedimentary basins, including the Neoproterozoic to Devonian Officer Basin (part of Centralian Superbasin)^21^ and the Mesozoic Bight and Cenozoic Eucla basins^22,23^. Aitken *et al*.^11^ correlated the Sabrina Sedimentary Basin and the Bight/Eucla basins based on the pre-Gondwanan breakup proximity. The Sabrina Sedimentary Basin is not exposed, although, the presence of rare glaciogenic sedimentary material along the Wilkes Land coast^24–26^ provides a unique opportunity to study this basin.

In this paper, we analyse geological samples from crystalline basement and sedimentary cover in order to characterise part of the subglacial bedrock of interior Wilkes Land between the Wilkes Province and Mawson Craton. We combine new U–Pb–Hf isotopic data of coastal basement outcrops in Antarctica with the aeromagnetic signature of conjugate Australian-Antarctic basement domains to identify two new Mesoproterozoic basement provinces of Coompana and Madura affinity in Wilkes Land and resolve their geometry in their Rodinian and Gondwanan configurations. We then use detrital zircon U–Pb–Hf and authigenic monazite U–Th–Pb isotopic data from rare sandstone erratic samples to suggest the presence of Neoproterozoic sedimentary rocks in the Sabrina Sedimentary Basin and correlate this basin with the eastern Neoproterozoic Officer Basin. Our new interpretations of subglacial bedrock strengthen correlations between Antarctic and Australian geological counterparts and provide key constraints for global supercontinent reconstructions.

## U–Pb–Hf Geochronology of Chick Island

**Figure 2:**
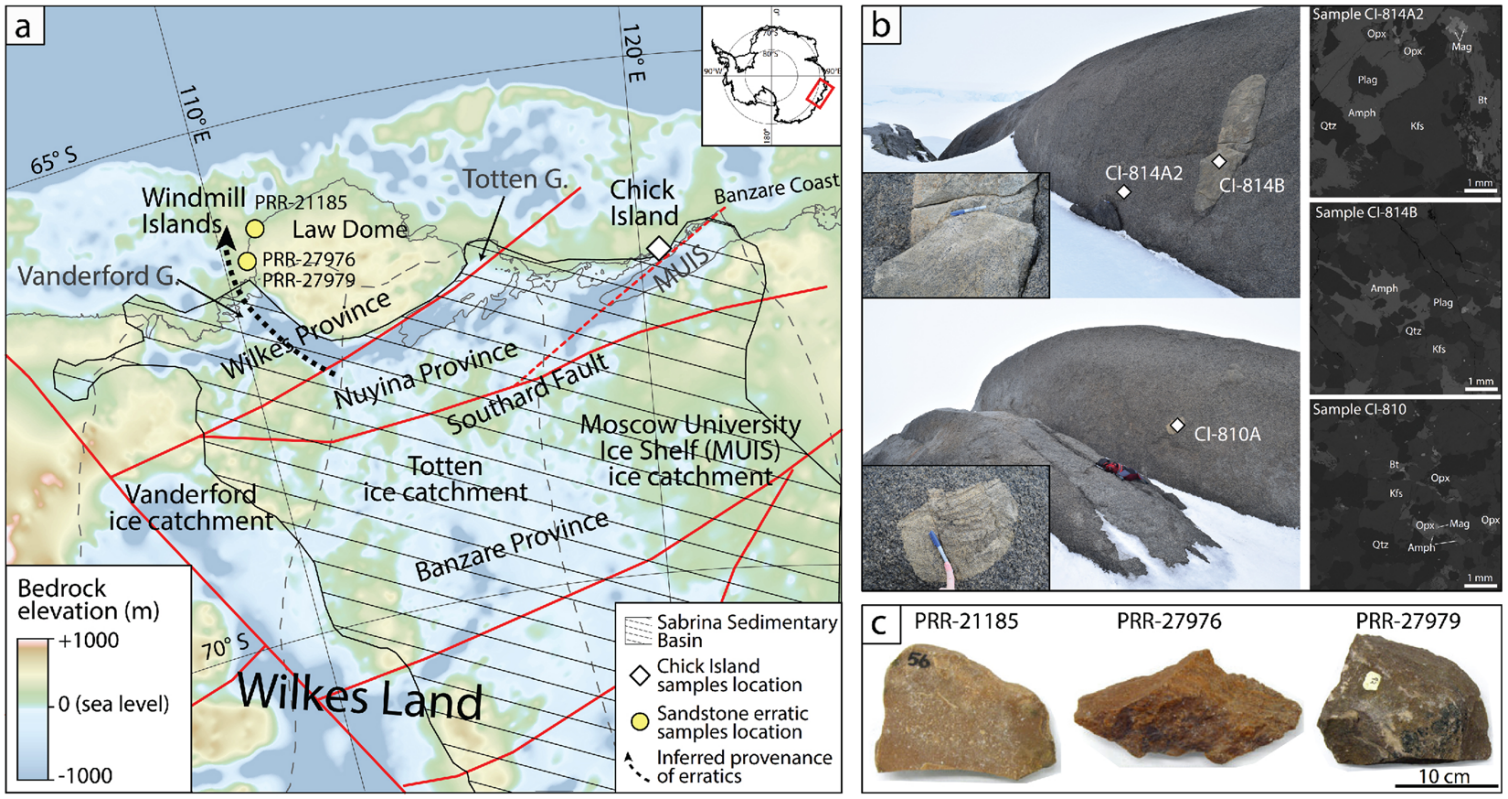
(**a**) Subglacial bedrock elevation map showing location of samples of this study, ice catchment basins^31^ (dashed black lines) and major structural lines^7^ (solid red lines); revised path of the Southard Fault is also shown as dotted red segment; (**b**) (left) Location of sampling sites at Chick Island and (right) annotated detail of backscatter electron (BSE) image of each sample; (**c**) glacial sandstone erratic samples used in this study. Abbreviations are: Amph–Amphibole; Bt–Biotite; Kfs–K-feldspar; Mag–Magnetite; Opx–Orthopyroxene; Plag–Plagioclase; Qtz–Quartz.

We analysed zircon from three igneous bedrock samples from the remote Chick Island outcrop east of the Totten Glacier and along the Banzare Coast (Fig. 2a), including one sample of the main pluton and two petrologically-similar xenolith samples (Fig. 2b), to provide constraints on the age and composition of Precambrian basement in this poorly exposed region.

**Figure 3:**
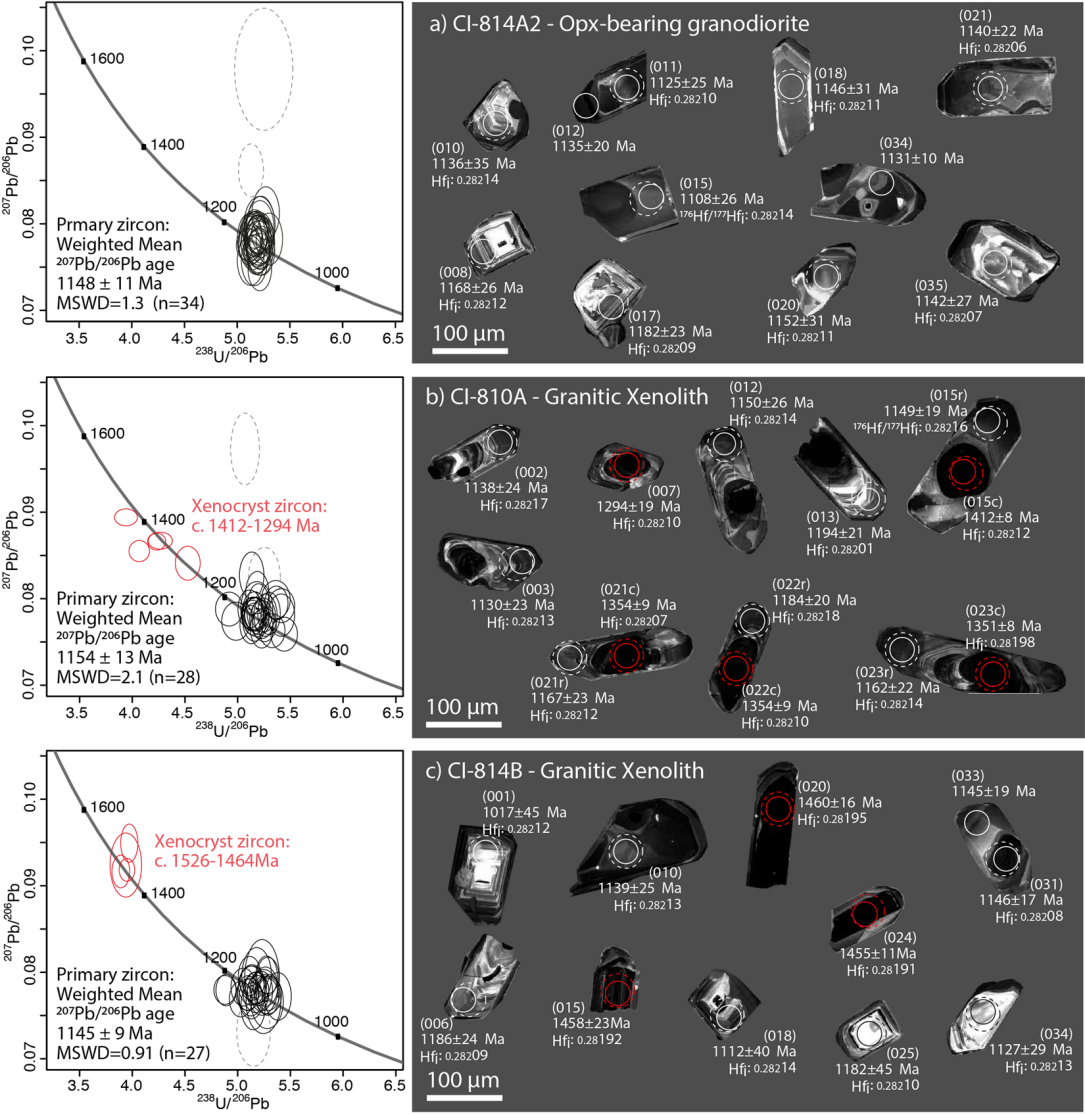
(left) U–Pb Tera-Wasserburg plots and (right) representative zircon CL images from each Chick Island sample. On Tera–Wasserburg plots, dashed grey ellipses denote analyses that have been excluded from age calculations on the basis of discordance (>10%). On CL images, U–Pb and Lu–Hf analysis locations are displayed as solid and dashed circles, respectively. Corresponding spot number (in brackets), apparent ^207^Pb/^206^Pb ages and initial ^177^Hf/^176^Hf ratio (Hf_i_) and are also given for each analysis. Solid red ellipses on Tera–Wasserburg plots and red circles on CL images correspond to xenocryst zircon analyses.

**Figure 4:**
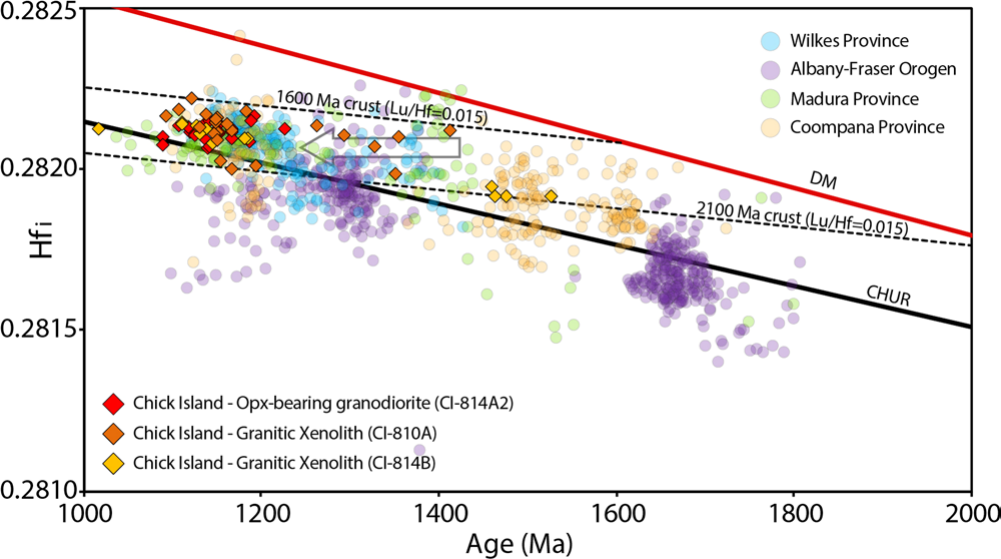
Initial ^177^Hf/^176^Hf ratios (Hf_i_) for concordant zircon from Chick Island samples. The ratios are plotted against the apparent ^207^Pb/^206^Pb age for each analysis. Hf_i_ of Chick Island zircon is compared to U–Pb–Hf data of igneous zircon from: Albany-Fraser Orogen (GSWA as compiled by Smits *et al*.^62^), Windmill Islands^8,9^, Coompana and Madura provinces^14^. Grey arrow indicates possible Pb-loss trajectory in xenocryst zircon from sample CI-814A. Zircons plotting below the CHUR (chondritic uniform reservoir) reference line reflect an increasingly crustal melt source, whereas those plotting at progressively more positive Hf_i_ values above CHUR reflect an increasing contribution from the depleted mantle (DM) to magmas from which the zircon formed. Crustal growth curves using a bulk crust value of ^176^Lu/^177^Hf = 0.015^63^ are drawn through the most evolved (c. 2100 Ma) and least evolved (c. 1600 Ma) Chick Island zircon.

Sample CI-814A2, the main rock type exposed at Chick Island, is a coarse-grained orthopyroxene-bearing granodiorite (based on Quartz-Alkali Feldspar-Plagioclase (QAP) modal classification of Streckeisen^27^) and contains quartz (~33%), K-feldspar (~19%), plagioclase (~43%) as well as biotite, orthopyroxene, amphibole, ilmenite and magnetite (~5%), with accessory apatite and zircon (Fig. 2b). Zircon grains extracted from this sample are 100–250 μm long and equant to elongate, with aspect ratios from 1:1–4:1 (Fig. 3a). Under cathodoluminescence (CL), zircon grains are medium to brightly luminescent, commonly displaying oscillatory (e.g. spot 008, 017) and/or sector (e.g. spot 021) zoning (Fig. 3a). Thirty-six U–Pb analyses were collected from 31 grains across the spectrum of internal domains observed under CL. However, regardless of zonation, concordant analyses yield a ^207^Pb/^206^Pb weighted average age of 1148 ± 11 Ma (n = 34, mean square of weighted deviates [MSWD] = 1.34), interpreted to represent the crystallisation age of this sample (Fig. 3a). These zircon grains possess a steep HREE pattern, positive Ce and negative Eu anomalies and a Th/U ratio (0.86–2.03) consistent with magmatic zircon (see Supplementary Fig. S1). Twenty-four Hf isotope analyses of concordant primary igneous zircon grains yield initial ^176^Hf/^177^Hf (Hf_i_) ratios in the range 0.28206–0.28216 (Fig. 4) and initial epsilon Hf (εHf_i_) values between −0.53 and +4.87. A single outlier (spot 019) yielding Hf_i_ ratio of 0.28101 and εHf_i_ of −36.74 was excluded from the interpretation.

Two xenoliths hosted in the granodiorite bedrock (samples CI-810A and CI-814B) have a granitic composition and contain igneous microstructures characterised by a lack of recrystallisation and the presence of interstitial igneous green amphibole. Samples contain quartz (~38%), K-feldspar (~24%), plagioclase (~33%) as well as amphibole and orthopyroxene (~5%) and accessory apatite and zircon (Fig. 2b). The apparent foliation observed at the outcrop scale is due to magmatic compositional banding into K-feldspar-rich versus plagioclase-rich domains that may reflect accumulation or magmatic flow processes.

Zircon grains in sample CI-810A are 100–200 μm long and have aspect ratios that vary from 1:2–1:4. Under CL (Fig. 3b), the majority of zircon grains contain dark resorbed cores with low-CL response (e.g. spots 023c, 021c) that are overgrown by moderate CL oscillatory zoned rims (e.g. spots 002, 021r) or more diffusely zoned rims (e.g. 015r). Thirty-five U–Pb analyses were collected from twenty-one zircon grains, including seven grains where both cores and rims were analysed. Rim domains represent the main concordant zircon population, which yields a ^207^Pb/^206^Pb weighted average age of 1154 ± 13 Ma (n = 28, MSWD = 2.1; Fig. 3b), within error of the interpreted age of crystallisation of the host granodiorite (sample CI-814A2). This c. 1154 Ma population possesses high (0.38–1.45) Th/U ratios, steep HREE patterns, positive Ce and negative Eu anomalies (Supplementary Fig. S1), consistent with those of primary igneous zircon in sample CI-814A2. Accordingly, we interpret this c. 1154 Ma population in the xenoliths to date cognate inclusions. Hf isotopic analyses of eighteen concordant zircon grains from the c. 1154 Ma zircon population yield Hf_i_ ratios in the range 0.28200–0.28222 (Fig. 4) and εHf_i_ values from −1.47 to +5.45. Five older analyses corresponding to dark CL cores reveal high concentrations of U (419–3565 ppm) with individual apparent ^207^Pb/^206^Pb ages spread along Concordia bet]ween c. 1412 Ma to c. 1294 Ma (Fig. 3b). Trace element ratios (Th/U) and REE patterns for these zircon cores are distinct from the primary igneous zircon population (Supplementary Fig. S1). Five core domains yield Hf_i_ ratios in the range 0.28198–0.28212 (Fig. 4) and εHf_i_ values from +2.05 to +8.30. We interpret this population to represent xenocrystic components, and the spread in apparent ages as the result of variable resetting of metamict zircon (alpha dose >8 α/mg × 10^15^) during post-crystallisation modification (Supplementary Table S2).

Zircon grains in sample CI-814B are 70–200 μm long and have aspect ratios that vary from 1:1–1:4. Many of the larger zircon grains show medium to high CL response (Fig. 3c) with oscillatory (e.g. spots 001, 006) or diffuse banded (e.g. spot 33) zoning. A subset of the grains contain low-CL domains that are typically unzoned (e.g. spot 020). Thirty-two analyses were collected from thirty-one grains representing the range of observed morphologies and zonation. The main concordant zircon population yields a ^207^Pb/^206^Pb weighted average age of 1145 ± 9 Ma (n = 27, MSWD = 0.91; Fig. 3c), with the majority of these grains exhibiting high (0.54–1.90) Th/U ratios and REE trace element signatures typical of igneous zircon (Supplementary Fig. S1). Hf isotopic analyses from the c. 1145 Ma zircon population yield Hf_i_ ratios in the range 0.28208–0.28214 (Fig. 4) and εHf_i_ values between −0.47 to +2.37. As for sample CI-814B, we interpret this population as indicating a cognate inclusion relationship for both xenoliths. Four older analyses on homogenous dark zircon domains yield apparent ^207^Pb/^206^Pb ages ranging from c. 1526 Ma to c. 1464 Ma, have slightly more REE-enriched compositions compared to the main population (Fig. 3c) and yield Hf_i_ ratios in the range 0.28191–0.28195 (Fig. 4) and εHf_i_ values between +2.17 to +3.64. We interpret this population to represent xenocrysts and suggest their clustered U–Pb–Hf signature fingerprints the character of early Mesoproterozoic (c. 1500 Ma) basement at depth.

## Basement Correlations Between Wilkes Land and Australia

Aeromagnetic signatures from the conjugate Australian-Antarctic margins suggest the presence of similar basement domains in the Coompana/Madura Provinces and Wilkes Land. In southern Australia, the Coompana Province has variable magnetic intensity^28^ and is characterised by large areas of low to moderate magnetic intensity (<−300 nT) associated with the c. 1610–1490 Ma plutons of the Toolgana and Undawidgi Supersuites (Fig. 1b). In contrast, the Madura Province exhibits overall higher-frequency anomalies with moderate to strong magnetic intensity (0–100 nT). The c. 1192–1125 Ma Moodini Supersuite in both the Coompana and Madura provinces exhibits a distinct NE directional trend and is the source of the highest magnetic intensities (up to 400 nT) in both provinces (Fig. 1b).

In Antarctica, we suggest that two large and magnetically distinct intrusive suites identified by Aitken *et al*.^11^ match, in type and character, the magnetic anomalies associated with the Undawidgi–Toolgana and Moodini supersuites (Fig. 1b). The older suite is characterised by strong relative magnetic lows, providing a good match with magnetic anomalies corresponding to the Toolgana and Undawidgi supersuites. The younger suite, is interpreted to occur throughout the entire region based on identical high magnetic character and north-easterly directional trend as c. 1150 Ma granitoids across southwestern Australia^11^ which crosscut the major regional structural grain. We suggest this suite is likely equivalent to the Moodini Supersuite granites.

The spatial distribution of these suites in Wilkes Land defines two distinct geophysical domains with different magnetic character: these are separated by a previously un-named ENE-WSW geophysical lineament^11^ that marks a sharp change in overall intensity and frequency of magnetic anomalies (Fig. 1b). We suggest that the presence of high-amplitude magnetic anomalies associated with Undawidgi–Toolgana and Moodini supersuites exclusively south of this lineament fingerprints a Coompana-type basement complex. In contrast, the overall relatively higher magnetic intensity and frequency of magnetic anomalies combined with the absence of a geophysical signal like that of the Undawidgi–Toolgana plutons indicates the presence of a Madura-type basement complex in the portion of Wilkes Land wedged between the ENE-WSW lineament and the Wilkes Province (Fig. 1b). We identify this ENE-WSW lineament as the boundary between Coompana- and Madura-type crust in Antarctica and name the Antarctic conjugates of the Coompana and Madura provinces the Banzare Province and Nuyina Province, respectively.

The Chick Island outcrop corresponds with an interpreted Moodini Supersuite pluton located approximately 100 km north of the inferred boundary between the Banzare and Nuyina provinces (Fig. 1a,b); our new U–Pb–Hf zircon data provide insight into its crustal affinity and a test of the tectonic correlation based on aeromagnetic data. The Chick Island granodiorite pluton (sample CI-814A2) has a crystallisation age of c. 1148 Ma and Hf_i_ isotopic values consistent with the emplacement age and Hf signature of the Moodini Supersuite in the Coompana and Madura provinces^14^, as well as isotopically-similar igneous rocks found in the Wilkes Province (e.g. Ardery Charnockite in the Windmill Islands^8^) (Fig. 4). Two cognate inclusions yield c. 1150 Ma primary zircon populations and xenocrystic zircon grains that fingerprint crustal contamination. The distinct Hf_i_ isotopic character of xenocrystic zircon suggests at least two isotopically different components in the source: the less radiogenic xenocrysts (c. 1526–1464 Ma; sample CI-814B) overlap in age and Hf signature with zircon from the Coompana Province, and the more radiogenic xenocryst group (c. 1412–1294 Ma; sample CI-810A), though displaying variable Pb loss possibly from c. 1420 Ma, overlap the isotopic signature of the more juvenile Madura Province (Fig. 4). We suggest that these U–Pb–Hf patterns are indicative of crustal contamination from both Coompana- (c. 1610–1490 Ma) and Madura-type (c. 1475–1389 Ma) crust at depth, and thus fingerprint the presence of a tectonic boundary between the Banzare and Nuyina provinces in Antarctica, with Chick Island being the most likely coastal location of this boundary.

Based on our new geological data, we revise the path of the tectonic boundary between the Banzare and Nuyina provinces identified in aeromagnetic data (ENE-WSW lineament of Aitken *et al*.^11^) to intersect the coast in the proximity of the Chick Island outcrop (dotted segment in Figs 1 and 2). We name this composite tectonic boundary the Southard Fault (after Cape Southard).

Correlations of the Coompana/Madura and Banzare/Nuyina provinces across Australia-Antarctica are offset by approximately 330 km sinistral strike-slip motion along the Mundrabilla-Frost Shear Zone (Fig. 1). To reconstruct the configuration of these provinces prior to this motion, we link the Southard Fault with a major NE-trending structure identified in aeromagnetic data by Aitken *et al*.^11^, which we suggest may represent the conjugate boundary between the Coompana and Madura provinces in Australia (Fig. 1c,d). Despite the lack of geological data from this region, this structure appears to represent the southerly expression of the boundary between the West Musgrave and central Musgrave provinces which preserve evidence of tectono-magmatic affinity with the Madura and Coompana provinces, respectively^7,29^ (Fig. 1c,d). This connection allows the Madura Province to continue across the Mundrabilla-Frost Shear Zone in Australia and resolves the geometry of Coompana and Madura provinces and the conjugate Banzare and Nuyina provinces before and after strike-slip motion in their Rodinia (c. 1130–1040 Ma) and Gondwana (c. 160 Ma) configurations.

## Provenance and Age of Sandstone Erratics

**Figure 5:**
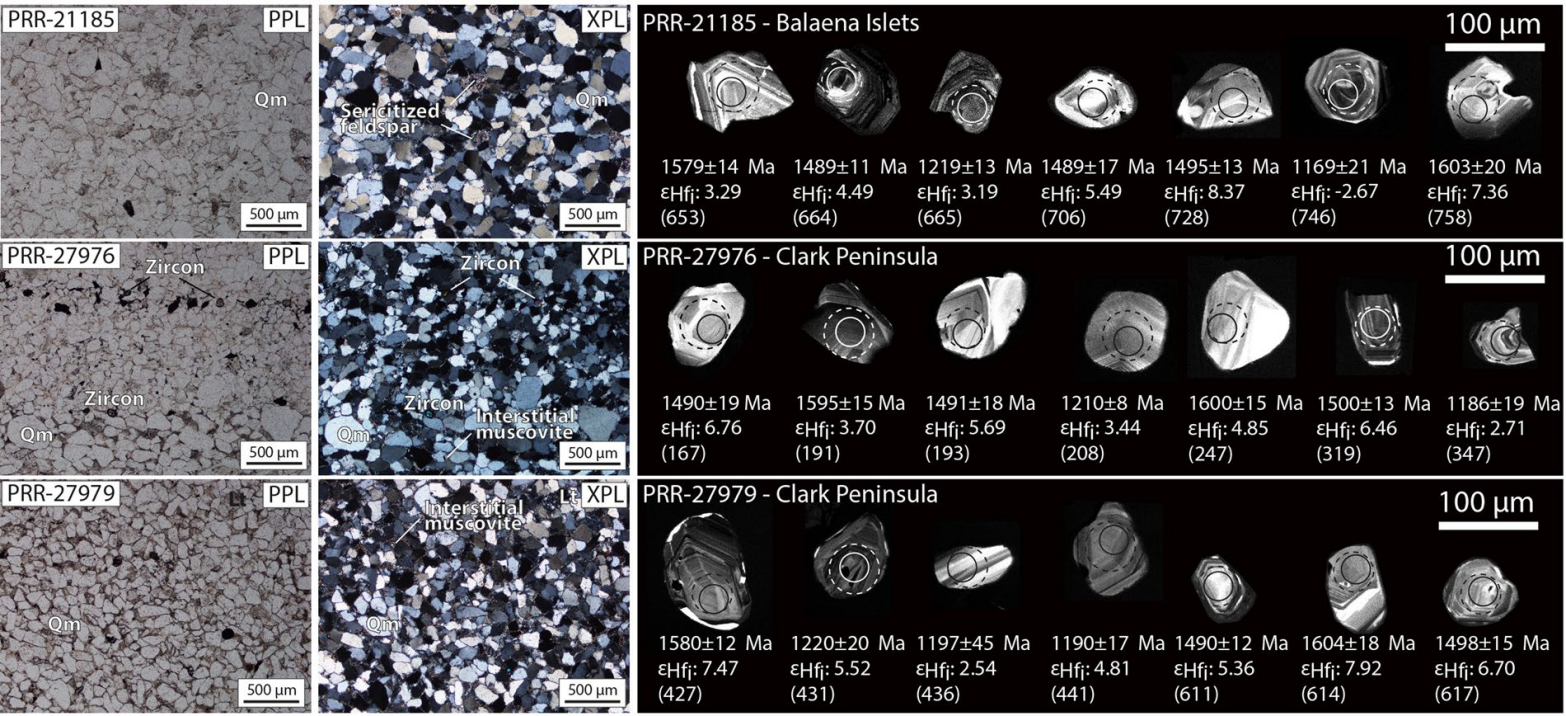
(left to right) Plane-polarised (PPL) and cross-polarised (XPL) microphotographs with descriptive labels, and CL images of representative detrital zircons used for U–Pb–Hf dating from each sandstone erratic sample. On CL images, U–Pb and Lu–Hf analysis locations are displayed as solid and dashed circles, respectively. Corresponding apparent ^207^Pb/^206^Pb ages (Ma), initial epsilon Hf (εHf_i_) and spot number (in brackets) are also shown for each analysis. Abbreviations are: Lt–Lithic fragment; Qm–Quartz monocrystalline.

To probe the provenance and age of sedimentary rocks overlying crystalline basement in interior Wilkes Land, we analysed three glacially-transported sedimentary samples (Fig. 2c) collected at two localities in the Windmill Islands^26^ (Fig. 2a) by combining detrital zircon and authigenic monazite dating. The glacial erratics are faceted and angular, suggesting these samples were derived from a proximal sedimentary source (Fig. 2a). Samples are well sorted quartz-arenites (Fig. 5) dominated by monocrystalline quartz (>95%, derived from point counting) and small proportions of K-feldspar (up to 3%). This composition suggests a dominantly granitic sediment source and a “craton interior” tectonic provenance for the three samples. The presence of fine-grained (~10 μm) interstitial muscovite and sericitised feldspar grains (Fig. 5) indicates post-depositional interaction with hydrothermal fluids.

**Figure 6:**
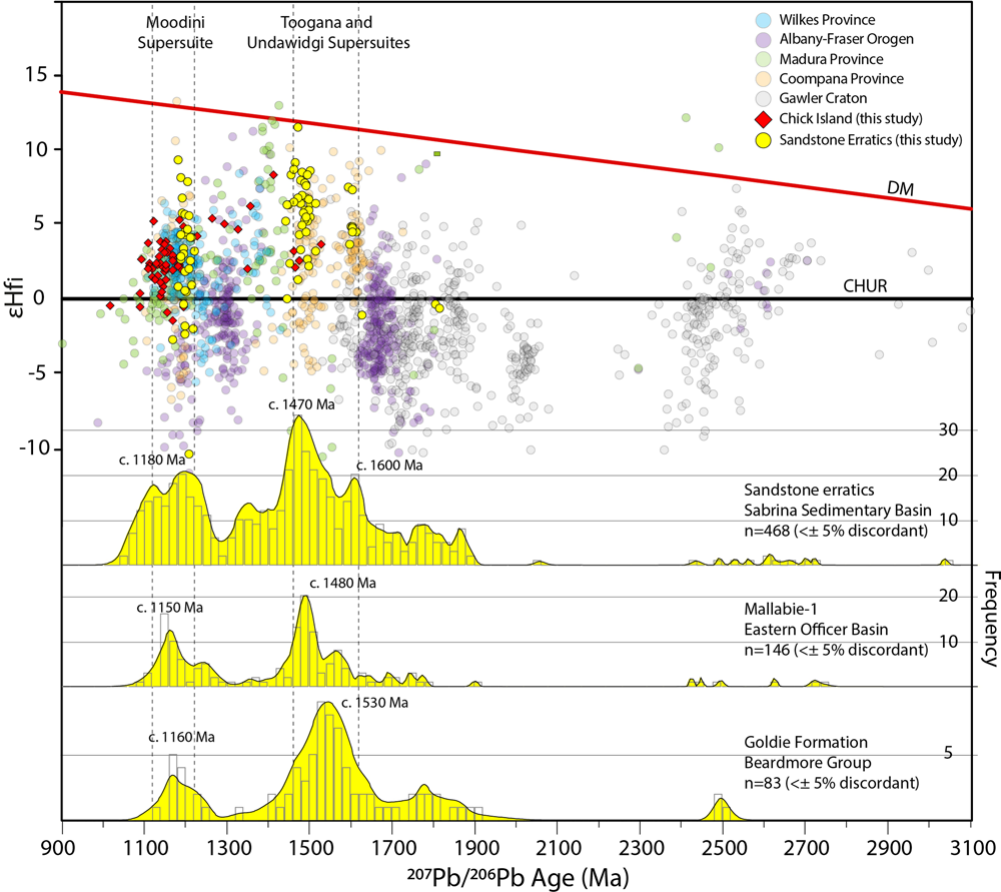
Initial epsilon Hf (εHf_i_) values (yellow dots) versus probability density plot (yellow fill) and histograms for detrital zircon from the sandstone erratic samples. εHf_i_ ratio is compared to the same U–Pb–Hf data compilation used in Fig. 3 with the addition of >1500 Ma zircon from Gawler Craton^37^. Composite probability density plots and histograms for the Mallabie-1 sedimentary samples (Officer Basin)^38^ and the Goldie Formation (Beardmore Group)^36^ are also shown for comparison.

Heavy liquid separation of 100 g of crushed material from each of the three samples provided a total of ~1000 zircon grains in the size range of 20–140 μm. The size fraction of the analysed zircons ranges from ~40–120 μm. Zircons are sub-rounded to sub-euhedral, with the majority of grains (~80%) having aspect ratios from 1:2–1:3 and indicating an overall local sourcing of sediments. U–Pb zircon ages from the three samples reveal similar age peaks and have a statistically similar detrital distribution according to the performed Kolmogorov–Smirnov (K-S) test (maximum difference between age distribution curves [D] = 0.0891–0.1471 and p-values > 0.05) (see Supplementary Fig. S2). The composite detrital zircon age spectrum, derived from a total of 468 concordant (<± 5% discordant) U–Pb zircon analyses from the three samples, indicates sedimentary source regions dominated by c. 1600–1470 Ma (48% of total) and c. 1180 Ma zircons (34%), with a lower proportion of zircons in the age range from c. 2400 Ma to 1700 Ma (18%) (Fig. 6). Most zircon grains in the two main groups display oscillatory zoning (Fig. 5) and average Th/U of ∼0.64, typical of igneous zircon. An estimate of the maximum deposition age for the sandstones is 1091 ± 7 Ma (n = 40, MSWD = 1.4), based on the youngest detrital zircon population. The zircon εHf_i_ data from the sandstone erratic samples are dominated by positive values in each of the main detrital populations reflecting a significant mantle contribution in the source magmas. The c. 1600–1470 Ma zircons have overall positive εHf_i_ values between +11.52 and −1.08, while the c. 1180 zircons have a range of εHf_i_ values between +9.34 to −2.67 (Fig. 6).

**Figure 7:**
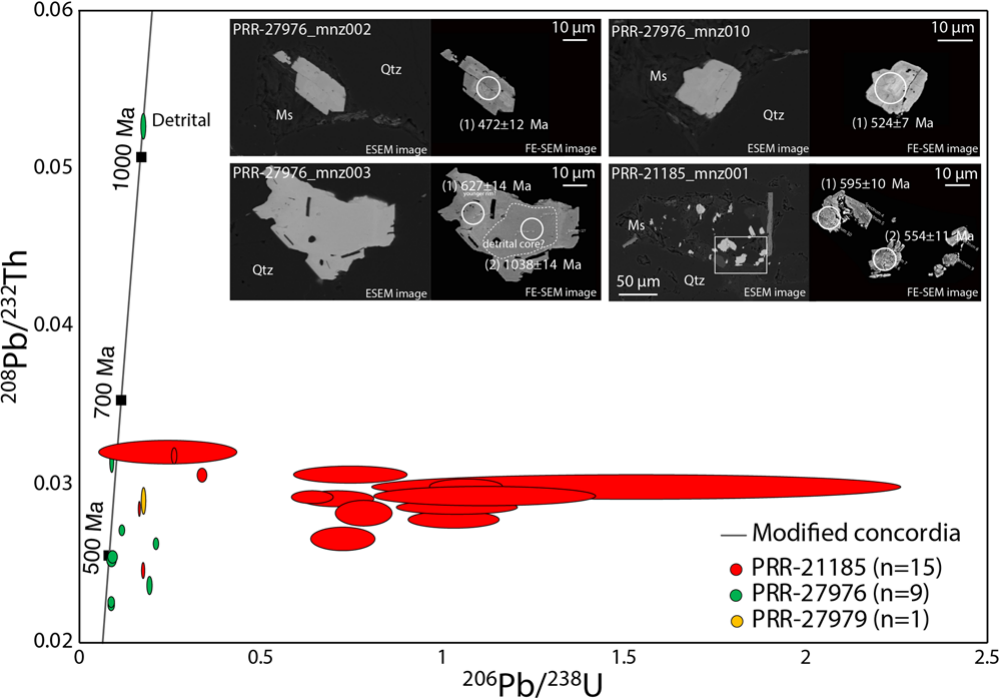
^206^Pb/^238^U vs ^208^Pb/^232^Th modified concordia diagram with twenty-five analyses obtained by *in situ* U–Th–Pb dating for twenty-one monazite grains. Data-point error ellipses are 1σ; (inset) annotated ESEM image (left) and FE-SEM image (right) of four representative monazite grains used for *in situ* U–Th–Pb dating. Sample and grain number are indicated for each image. U–Th–Pb spots are 9 μm; spot numbers are shown in brackets; apparent ages are reported as ^208^Pb/^232^Th ages; Abbreviations are: Ms–Muscovite; Qtz–Quartz.

Rare monazite typically occurs as irregular grains (~15–50 μm) amongst the fine-grained interstitial muscovite (Fig. 7 inset). This texture, together with the relatively high Th (mostly ~10,000 ppm) and low U (mostly <100 ppm), suggests the analysed monazite grains are authigenic and likely formed due to an interaction with hydrothermal fluids during or after deposition^30^. A total of 32 grains were analysed from the three sandstone erratic samples. Seven analysis were discarded due to low analytical signal intensity for the U, Th and Pb isotopes. U/Pb systems in analysed monazite grains are overall disturbed due to common Pb contamination and depletion of U relative to Th, resulting in overestimated ^206^Pb/^238^U ages and large analytical uncertainties (Fig. 7). The well-resolved ^208^Pb/^232^Th ages obtained confirm instead minor disturbance of the Pb/Th systems and provide more reasonable age estimates.

Fifteen authigenic monazite grains analysed in sample PRR-21185 yield apparent ^232^Th/^208^Pb ages ranging between c. 639–490 Ma (Fig. 7). Eight authigenic monazite analyses from sample PRR-27976 yield ages ranging between c. 627–449 Ma (Fig. 7). A ^232^Th/^208^Pb apparent age of c. 1038 Ma obtained for one older monazite grain core exhibiting relatively lower Th (4282 ppm) and higher U (1664 ppm) is interpreted as a detrital relict. A single analysis obtained from sample PRR-27979 yielded an apparent ^232^Th/^208^Pb age of 577 ± 17 Ma (Fig. 7).

The observed spread in ^232^Th/^208^Pb ages could indicate different stages of authigenic monazite formation during the Late Neoproterozoic-Cambrian; we suggest that the oldest analyses in samples PRR-21185 and PRR-27976 may be closer to the depositional age of the sandstone while the apparently younger analyses record subsequent generation(s) of post-depositional authigenic monazite formation/hydrothermal alteration. An age of c. 633 Ma (average of two oldest ^232^Th/^208^Pb ages in samples PRR-21185 and PRR-27976) is therefore considered a minimum deposition age for the sandstone samples. The age of the single detrital monazite core also further constrains the maximum deposition age of the sandstones to 1038 ± 16 Ma.

## A neoproterozoic Sedimentary Basin in Interior Wilkes Land

New zircon U–Pb–Hf isotopic data from sandstone erratic samples allowed us to investigate, for the first time, the age and provenance of the enigmatic Sabrina Sedimentary Basin, interpreted to cover vast tracts of interior Wilkes Land (Figs 1a and 2a). Present-day ice divides and ice-flow directions^31,32^ indicate that the sandstone erratics were likely eroded from inland areas of the Vanderford ice catchment and transported to their present locations during a former expansion of the Vanderford Glacier^24,25^ (Fig. 2a). To our knowledge, these sandstone erratics represent the first samples of the Sabrina Sedimentary Basin ever to be examined in detail.

The Sabrina Sedimentary Basin has been interpreted as the Antarctic equivalent of Australian Mesozoic-Cenozoic break-up sedimentary basins^11^. However, U–Pb dating of detrital zircon and *in-situ* authigenic monazite constrain a much older Neoproterozoic (c. 1038–633 Ma) depositional age range for at least part of the basin. This is further supported by the lack of Pan-African aged (c. 600–500 Ma) detrital zircon which instead are consistently present in younger Australian Palaeozoic to Cenozoic sedimentary deposits^33,34^. This age range broadly corresponds to the deposition of the Neoproterozoic sedimentary sequences in the eastern Officer Basin^35^ of southern Australia (Fig. 1a), as well as Neoproterozoic sedimentary rocks of the Beardmore Group in the Transantarctic Mountains^36^. We therefore suggest that sedimentary cover similar in age and composition to the Neoproterozoic eastern Officer Basin and Beardmore Group occupies an area of approximately 100,000 km^2^ in the Vanderford ice catchment, and could extend over large parts of the Sabrina Sedimentary Basin further east.

The detrital signature of the sandstone erratics is defined by a broadly bimodal distribution: (1) the c. 1600–1470 Ma detrital zircon age and εHf_i_ signatures match the character of igneous zircons from the Toolgana and Undawidgi supersuites in the Coompana Province (Fig. 6), and (2) the c. 1180 Ma detrital zircon population is broadly similar in age and εHf_i_ to igneous zircons of the Moodini Supersuite of the Coompana and Madura provinces^14^, and Chick Island (this study), as well as isotopically-similar granitic rocks found in the Wilkes Province (e.g. Ardery Charnockite^8^; Fig. 6). We suggest granitic source rocks of these ages and isotopic compositions contribute to the main detrital zircon signature in Neoproterozoic sedimentary rocks in the Sabrina Sedimentary Basin and indicate a predominantly local derivation of sediments from the Banzare and Nuyina provinces and/or Australian equivalent basement provinces. Older age components between c. 1700 Ma and 2400 Ma, correlate with the age of known magmatic events in the Gawler/Mawson cratons^37^ (Fig. 6) and could be interpreted to indicate minor sedimentary input from the east.

In Australia, the southerly extension of the eastern Officer Basin in the Coompana and Madura provinces is poorly known and concealed by the Mesozoic and Cenozoic cover of the Bight and Eucla basins. Two sandstone samples from the Mallabie-1 drill hole in the Coompana Province are tentatively linked to the eastern Officer Basin and yield similar age spectra to our sandstone erratic samples^38^ (Fig. 6). While we note some differences in the dominant peak ages in the zircon age spectra between these Officer Basin sedimentary rocks and our samples, the overall detrital zircon age spectra signatures in the former also suggests a strong influence of proximal sediment contributions (i.e. Coompana Province and Musgrave Province)^39^. A similar provenance is also shared by parts of the Neoproterozoic Beardmore Group (i.e. Goldie Formation) which is also interpreted to reflect a dominantly Mesoproterozoic sediment source form interior Wilkes Land^36^.

## Implications of Subglacial Geology

Our new zircon U–Pb–Hf and monazite U–Th–Pb geochronology, combined with regional age data and aero-geophysical observations have allowed us to interpret the age and composition of bedrock in interior Wilkes Land and test tectonic models.

We provide the first geological evidence for the Antarctic counterparts of the Mesoproterozoic Coompana and Madura provinces, previously only documented in southern Australia and resolve the geometry of these conjugate Mesoproterozoic basement provinces in both a Rodinia and Gondwana configuration. The addition of these two new Antarctic provinces confirm the presence of a progressively westerly-younging sequence of juvenile magmatic arc-related rocks retreating away from the Mawson Craton and provides additional evidence for a subduction-driven Mesoproterozoic evolution of Australo-Antarctica as supported by recent tectonic models^7^. The improved geometrical correlation between basement provinces of Wilkes Land and southern Australia will be fundamental in further understanding the Mesoproterozoic evolution of Australo-Antarctica and as such, inform plate tectonic models for the amalgamation of Nuna and Rodinia and the configuration of Gondwana.

New zircon and monazite data from sandstone erratics demonstrate the presence of Neoproterozoic sedimentary rocks in the Sabrina Sedimentary Basin that are equivalent in age and provenance to the eastern Officer Basin of Australia and Beardmore Group in the Transantarctic Mountains. The occurrence of sedimentary rocks of this age was previously unknown for this sector of East Antarctica and has broader implications for understanding the Rodinia-to-Gondwana transition during the Neoproterozoic. Similarities in age and provenance between our sandstone erratics and the Beardmore Group in the Transantarctic Mountains suggest that Neoproterozoic sedimentary rocks may have once covered a large sector of East Antarctica, forming an extensive platform basin on the proto-pacific rifting Rodina margin^36^. However, due to the limited geological samples available, we are unable to define the extent of the newly identified Neoproterozoic Sabrina Basin outside the confines of the Vanderford ice catchment region. It therefore remains unclear whether the Neoproterozoic Sabina Basin formed an Antarctic extension of the larger Centralian Superbasin or constitutes a different Neoproterozoic Antarctic basin sharing provenance similarities with the Officer Basin. Furthermore, we do not exclude the presence of a younger Mesozoic or Cenozoic basin phase in the Sabrina Sedimentary Basin as previously hypothesised by Aitken *et al*.^11^, which could overlie older sedimentary sequences as seen in southern Australia with the overlap of the Bight and Eucla basins over the Officer Basin.

Our improved geological correlation between southern Australia and Wilkes Land, and interpretation of age and composition of subglacial bedrock, can also help inform estimations of the spatial distribution of crustal radiogenic heat production^40^ and geothermal heat flow^41^ across the Australian-Antarctic margin, with potential implications for ice sheet models that simulate past and future Antarctic ice sheet behaviour.

## Methods

### Zircon sample preparation and U–Pb–Hf analyses

Location of samples used in this study is given in Supplementary Table S1. Igneous zircon grains were separated from crushed rocks using a standard plastic pan and warm water and subsequent magnetic separation. Detrital zircon were separated from crushed rocks using heavy liquid separation. Grains were mounted in 25-mm diameter epoxy discs. Mounts were polished to half grain thickness to expose grain centres, carbon coated and imaged using a cathodoluminescence (CL) detector on a FEI Quanta 600 Environmental Scanning Electron Microscope (ESEM) at Central Science Laboratory, University of Tasmania, to identify compositional domains for analysis.

U–Pb zircon ages were collected at the University of Tasmania, Australia using laser ablation-inductively coupled plasma-mass spectrometry (LA-ICP-MS). U–Pb zircon analyses were performed in two different sessions on an Agilent 7900cs quadrupole ICPMS with a 193 nm Coherent Ar–F gas laser and a Resonetics S-155 ablation cell at the School of Earth Sciences, University of Tasmania. Each analysis was pre-ablated with 5 laser pulses to remove any surface contamination then the blank gas was analysed for 30 s followed by 30 s of zircon ablation at 5 Hz and 2 J/cm^2^ on a spot size of 29 μm. Elements measured include ^49^Ti, ^56^Fe, ^90^Zr, ^178^Hf, ^202^Hg, ^204^Pb, ^206^Pb, ^207^Pb, ^208^Pb, ^232^Th and ^238^U with each element being measured sequentially every 0.16 s with longer counting time on the Pb isotopes compared to the other elements. The international glass standard NIST610 was ablated at the beginning and end of the analytical session to correct for mass bias on the ^207^Pb/^206^Pb ratio. Each run consisted of 30–50 analyses of our unknowns, bracketed by 4–6 analyses of the primary reference zircon standard 91500^42^ used to correct for mass bias, machine drift and down-hole fractionation on the Pb/U and Pb/Th ratios, and 4 analyses (two each) of secondary standards TEMORA 1^43^ and GJ-1^44^/Plesovice^45^ to provide an independent control to assess accuracy and precision. Full tabulation of U–Pb isotopic data of unknowns and standards is reported in Supplementary Table S2. Data reduction calculations and error propagations were done with Microsoft Excel® via macros designed at the University of Tasmania using the techniques outlined by Sack *et al*.^46^. The degree of metamictisation was also determined in igneous zircon by using U and Th concentrations and ^207^Pb/^206^Pb ages to calculate the dose of α-events for each zircon grain. Zircon grains were classified as ‘highly crystalline’ when alpha dose was <3 α/mg × 10^15^, ‘moderately damaged’ when >3 and < 8 α/mg × 10^15^ and ‘highly metamict’ when >8 α/mg × 10^15^^47^. U–Pb Tera-Wasserburg plots of igneous zircons were constructed using isoplotR^48^. Error ellipses on Tera-Wasserburg plots are calculated at the two-sigma level. ^207^Pb/^206^Pb data are used for all age determinations. The quoted analytical uncertainties on individual analyses are given at the 2r level. Weighted mean ages are calculated to their 95% confidence level using analyses that are ≤±10% discordant (within 2r uncertainty of concordia). Probability density plots (PDP) of detrital zircon were calculated with DensityPlotter^49^ using ^207^Pb/^206^Pb ages and their 1σ uncertainties for data ≤±5% discordant. Histogram bin size in all PDPs is 20 Myr. Two-sample Kolmogorov–Smirnov (K-S) tests were performed on the detrital zircon age data using the online statistics calculator of Kirkman^50^ to determine if the samples were derived from different sources by comparing the distance between the cumulative age distribution curves.

Lu–Hf isotope analyses were performed on a subset of zircon grains already analysed for U–Pb using a New Wave/Merchantek LUV213 laser-ablation microprobe, attached to a Nu Plasma multi-collector inductively coupled plasma mass spectrometer (LA-MC-ICPMS) at GEMOC, Macquarie University (Sydney, Australia). Griffin *et al*.^51^ describe the methodology in detail. A blank gas was analysed for 60 s followed by 120 s of ablation at 5 Hz and 2 J/cm^2^ and a beam diameter of 40–50 µm (depending on the size of the zircon grain). Zircon CL images were used to ensure that Hf isotope analyses were contained within the same domain analysed for U–Pb. Our samples were measured in two analytical sessions. Zircons from the Mud Tank carbonatite locality were analysed together with the samples in each session to monitor accuracy of the results. Most data and the mean ^176^Hf/^177^Hf value are within 2 standard deviations (SD) of the recommended value [0.282522 ± 42 (2σ)]^52^. Temora zircon was also run as an independent check on the accuracy of the Yb correction. Temora zircon has an average ^176^Yb/^177^Hf ratio of 0.04, which is similar to the mean ^176^Yb/^177^Hf ratio of zircon in this study. The average ^176^Hf/^177^Hf ratio for Temora is consistent with the published value for the Temora standard [0.282687 ± 24 (2σ)^53^]. The initial ^176^Hf/^177^Hf (Hf_i_) value in zircon is calculated using the measured ^176^Hf/^177^Hf and apparent ^207^Pb/^206^Pb age. Calculation of εHf_i_ values employed the decay constant of Scherer *et al*.^54^ of 1.865 × 10^–11^. Full tabulation of zircon Hf isotopic data of unknown and standards is presented in Supplementary Table S3. Initial ^176^Hf/^177^Hf and εHf_i_ plots were constructed in Microsoft Excel®; zircon U–Pb and Hf isotopes from our samples are compared to a compilation of Australian and Antarctic isotopic data (provided in Supplementary Table S4).

### Monazite sample preparation and *in situ* U–Th–Pb analyses

Rock chips for *in situ* U–Th–Pb monazite dating were mounted with epoxy resin in ~12 mm thick x 25 mm diameter steel cylinder and polished using a 0.25 µm diamond polishing lap. Monazite grains were identified in rock chip laser mounts using Sparse Phase Liberation-Lite analysis (SPL-LT). Representative images of monazite grains selected for geochronology were taken using a FEI Quanta 600 SEM. Grains were also imaged using high contrast BSE imaging to detect zoning within the grains on a Hitachi SU-70 Field Emission Scanning Electron Microscope (FE-SEM).

Monazite grains from each of the three sandstone erratic samples were investigated for *in situ* U–Th–Pb analyses following the analytical procedures reported in Halpin *et al*.^55^ on the same LA-ICP-MS system used to collect U–Pb detrital zircon ages. Pre-ablation and ablation times follow the monazite methodology highlighted in Halpin *et al*.^55^ with operating conditions of 5 Hz and ∼2 J/cm^2^ on a spot size of 9 μm. Elements measured include ^27^Al, ^31^P, ^43^Ca, ^140^Ce, ^172^Yb, ^202^Hg, ^204^Pb, ^206^Pb, ^207^Pb, ^208^Pb, ^232^Th and ^238^U. The international glass standard NIST610 was used as a primary standard for trace element quantification assuming stoichiometric Ce in monazite and as a primary standard for the ^207^Pb/^206^Pb ratio correction factor for monazite. The down hole fractionation, instrument drift and mass bias correction factors for Pb/U and Pb/Th ratios on monazite grains were calculated using two analyses on the primary standard (14971-Mon–in-house standard) and one analysis on each of the secondary standard monazites RGL4B^56^, Bananeira^57^, and 94–222^58^ analysed at the beginning of the session and every 16–20 unknowns using the same spot size and conditions as used on the samples. Following the recommendations of Seydoux-Guillaume *et al*.^30^, Grand’Homme *et al*.^59^, ^208^Pb/^232^Th ages are preferred to the ^206^Pb/^238^U ages in all age determinations due to common Pb contamination and the relatively high Th/U ratio of monazite. Results are presented graphically in a ^206^Pb/^238^U vs ^208^Pb/^232^Th modified concordia plot. Uncertainties on individual spot ages are 1σ. Full tabulation of U–Th–Pb isotopic data of monazite unknowns and standards and modified concordia plot are reported in Supplementary Table S5. Data reduction calculations and error propagations were done with Microsoft Excel® via macros designed at the University of Tasmania using the techniques outlined by Sack *et al*.^46^ and Halpin *et al*.^55^. Age calculations. ^206^Pb/^238^U vs ^208^Pb/^232^Th modified Concordia diagrams were made in Microsoft Excel®.

### Aeromagnetic data interpretation and tectonic reconstructions

We use the most recent magnetic anomaly map of the Antarctic (ADMAP-2)^60^ and the comparable Australian dataset available from Geoscience Australia in conjunction with geological data to match the aeromagnetic signature of the Coompana/Madura provinces and interior Wilkes Land in the Gondwana full-fit (c. 160 Ma) plate reconstruction framework of Aitken *et al*.^7^ and identify broad basement domains and lineaments. Our interpretation of Antarctic aeromagnetic data builds on the most recent tectonic model of Wilkes Land of Aitken *et al*.^11^ and preliminary interpretation of the 2015 Coompana aeromagnetic survey^28^ based on the most recent drillhole data available from the Coompana Province. We reproduce the Late Mesoproterozoic reconstruction of the Rodinia configuration (c. 1130–1040 Ma) of Aitken *et al*.^7^ with reversal of approximately 330 km of sinistral offset on the Mundrabilla-Frost Shear Zone to reconstruct the geometry of the Madura/Coompana and Antarctic conjugate provinces across Australia-Antarctica.

## Supplementary information

Supplementary Figures S1-S2

Supplementary Table S1

Supplementary Table S2

Supplementary Table S3

Supplementary Table S4

Supplementary Table S5
