# Supplementary material for: Fingerprinting Proterozoic Bedrock in Interior Wilkes Land, East Antarctica
Source: Sci Rep. 2019 Jul 15;9:10192. doi: 10.1038/s41598-019-46612-y (PMC6629686; doi:10.1038/s41598-019-46612-y)
Supplement: Supplementary file 1 — Supplementary Figures S1-S2 [file 41598_2019_46612_MOESM1_ESM.docx]

**Fingerprinting Proterozoic Bedrock in Interior Wilkes Land, East Antarctica**

**Alessandro Maritati^1,*^, Jacqueline A. Halpin^1^, Joanne M. Whittaker^1^, and Nathan R. Daczko^2^**

^1^Institute for Marine and Antarctic Studies, University of Tasmania, Private Bag 129, TAS 7001, Australia

^2^ARC Centre of Excellence for Core to Crust Fluid Systems and GEMOC, Department of Earth and Planetary Sciences, Macquarie University, NSW 2109, Australia

*Correspondence to alessandro.maritati@utas.edu.au

Supplementary Files:

Supplementary Figure S1 – chondrite-normalized rare earth element (REE) spider diagrams and Th/U ratios of zircon from Chick Island samples (page 2 of this document).

Supplementary Figure S2 – detrital zircon probability density plots for each sandstone erratic sample and results of the two-sample Kolmogorov-Smirnov test (page 3 of this document).

Supplementary Table S1 – summary of samples location in separate Excel (.xlsx) file.

Supplementary Table S2 – new U-Pb zircon geochronology in separate Excel (.xlsx) file. Supplementary Table S3 – new zircon Hf isotopic data in separate Excel (.xlsx) file.

Supplementary Table S4 – compilation of zircon U-Pb-Hf isotopic data in separate Excel (.xlsx) file.

Supplementary Table S5 – new in situ U-Pb monazite geochronology in separate Excel (.xlsx) file.


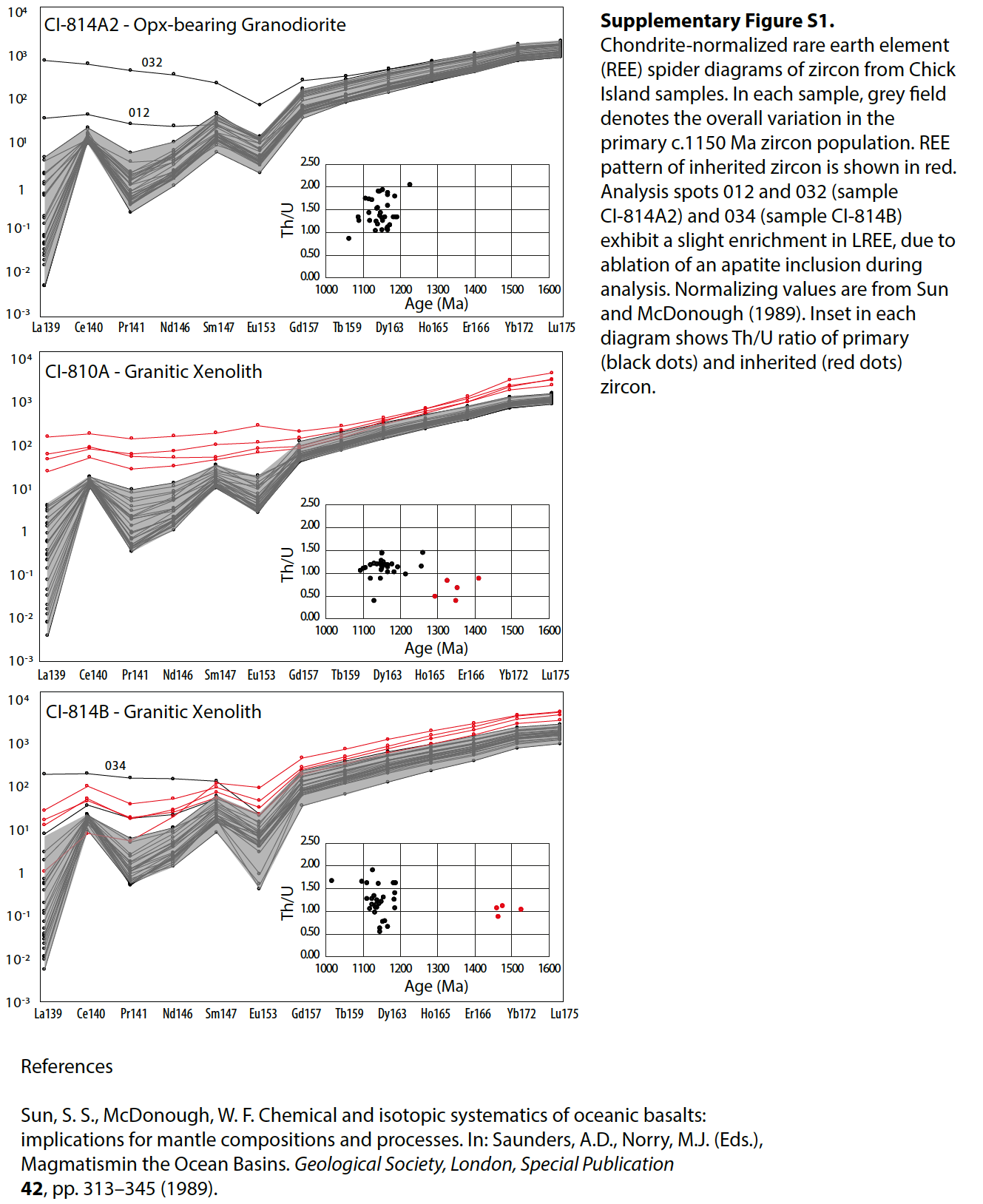

References

Sun, S. S., McDonough, W. F. Chemical and isotopic systematics of oceanic basalts: implications for mantle compositions and processes: In: Saunders, A. D., Norry, M. J. (Eds), Magmatism in the Ocean Basins. *Geological Society, London, Special Publication* **42**, pp. 313-345 (1989)
